# Cost-Effectiveness of Dipeptidylpeptidase-4 Inhibitors Added to Metformin in Patients With Type 2 Diabetes in China

**DOI:** 10.3389/fendo.2021.684960

**Published:** 2021-08-13

**Authors:** Wen-Qiang Lin, Zhong-jie Cai, Tingting Chen, Mao-Bai Liu, Na Li, Bin Zheng

**Affiliations:** ^1^Department of Pharmacy, Fujian Medical University Union Hospital, Fuzhou, China; ^2^Department of Pharmacy, Mindong Hospital of Ningde City, Fu’an, China; ^3^The School of Pharmacy, Fujian Medical University, Fuzhou, China

**Keywords:** dipeptidylpeptidase-4 inhibitors, cost-effectiveness, type 2 diabetes mellitus, incremental cost-effectiveness ratio, quality-adjusted life-year

## Abstract

**Purpose:**

Dipeptidylpeptidase-4 (DPP-4) inhibitors, including linagliptin, alogliptin, saxagliptin, sitagliptin, and vildagliptin, are used for the treatment of type 2 diabetes mellitus (T2DM) patients in China. This study assessed the economic outcomes of different DPP-4 inhibitors in patients with T2DM inadequately controlled with metformin in the Chinese context.

**Materials and Methods:**

In this study, the validated Chinese Outcomes Model for T2DM (COMT) was conducted to project economic outcomes from the perspective of Chinese healthcare service providers. Efficacy and safety, medical expenditure, and utility data were derived from the literature, which were assigned to model variables. The primary outputs of the model included the lifetime costs, quality-adjusted life years (QALYs), and incremental cost-effectiveness ratio (ICER). One-way and probability sensitivity analysis was conducted to assess the potential uncertainties of parameters.

**Results:**

Of the five competing strategies, alogliptin 25 mg strategy yielded the most significant health outcome, which associated with improvements in discounted QALY of 0.007, 0.014, 0.011, and 0.022 *versus* linagliptin 5 mg, saxagliptin 5 mg, sitagliptin 100 mg and vildagliptin50 mg, respectively. The sitagliptin 100 mg strategy was the cheapest option. The ICER of alogliptin 25 mg against sitagliptin 100 mg strategy was $6,952 per additional QALY gained, and the rest of the strategies were dominated or extended dominated. The most influential parameters were the cost of DPP-4 inhibitors and their treatment efficacy.

**Conclusions:**

These results suggested that alogliptin was a preferred treatment option compared with other DPP-4 inhibitors for Chinese patients whose T2DM are inadequately controlled on metformin monotherapy.

## Introduction

Type 2 diabetes is a chronic disease that leads to significant morbidity and mortality in poorly controlled patients (1). According to the international diabetes federation, 1 in 11 adults has diabetes. This equals 424.9 million people at a global level (2). The loss of productivity due to diabetes and its health consequences imposes an economic burden on patients, healthcare providers, and national economies. The World Health Organization (WHO) estimates that the cost of lost productivity for diabetic patients may exceed five times the direct cost of the disease (3). According to statistics, the economic burden caused by diabetes in 2018 reached 1.8% of global gross domestic product (GDP) and 12% of global health expenditures (4). In addition, more than 80% of the deaths caused by diabetes each year occur in developing countries, so the economic burden of these countries is more serious than that of developed countries (5). China also has a huge disease burden related to diabetes; the financial burden caused by diabetes on the Chinese economy increased from 2.216 billion Chinese yuan in 1993 to 200 billion Chinese yuan in 2007 (6, 7). Based on Chinese Type 2 Diabetes Prevention Guidelines (8), metformin is used as the first-line drug. There is a debate about the best second-line therapy after the failure of metformin monotherapy due to the increasing availability of antidiabetic drugs and the lack of comparative clinical trials of secondary treatment options. Sulfonylureas (SU) are a common second-line treatment because they have a fast onset of hypoglycemic effect (9). However, there are safety-related issues, including the risk of hypoglycemia and weight gain (9). In contrast, newer drug classes [e.g., dipeptidylpeptidase 4 (DPP-4) inhibitors] have shown clinical benefits in combination therapy (10).

By inhibiting the DPP-4 enzyme, which rapidly degrades two major incretin hormones, glucagon-like peptide-1 (GLP-1) and glucose-dependent insulinotropic polypeptide, the DPP-4 inhibitors have been approved as the recent class of therapies for managing T2DM (11, 12). In several clinical trials with T2DM, DPP-4 inhibitors, such as linagliptin, alogliptin, saxagliptin, sitagliptin, and vildagliptin, are found to decrease HbA1c, fasting plasma glucose (FPG) levels, and body weight in a variety of designs involving monotherapies and combination therapies (13). In China, linagliptin, alogliptin, saxagliptin, sitagliptin, and vildagliptin have been approved for treating T2DM. According to the expert consensus on the clinical application of DPP-4 inhibitors in China (14), these five types of DPP-4 inhibitors combined with metformin have little difference in clinical indications. They can all be used in combination with diet and exercise therapy for type 2 diabetes patients who still have poor blood glucose control after metformin monotherapy or are receiving the combined treatment of the two. As a chronic and progressive disease, the financial burden of T2DM over the long run is considerable, so it is necessary to provide a reference basis for health policy makers to use health resources rationally through decision analysis. Previously, there have been economic evaluations of DPP-4 compared to other antidiabetic agents [e.g., sodium-glucose cotransporter-2 (SGLT2) inhibitors, GLP-1 receptor agonists, and SU] (15, 16). However, there have been only a paucity of reports of head-to-head cost-effectiveness evaluations of DPP-4 inhibitors. This study aims to provide cost-effectiveness evidence for the use of different DDP-4 inhibitors to treat adult patients with T2DM uncontrolled on background metformin therapy by using the Chinese Outcomes Model for T2DM (COMT) (17, 18).

## Methods

### Model Overview

**Figure 1:**
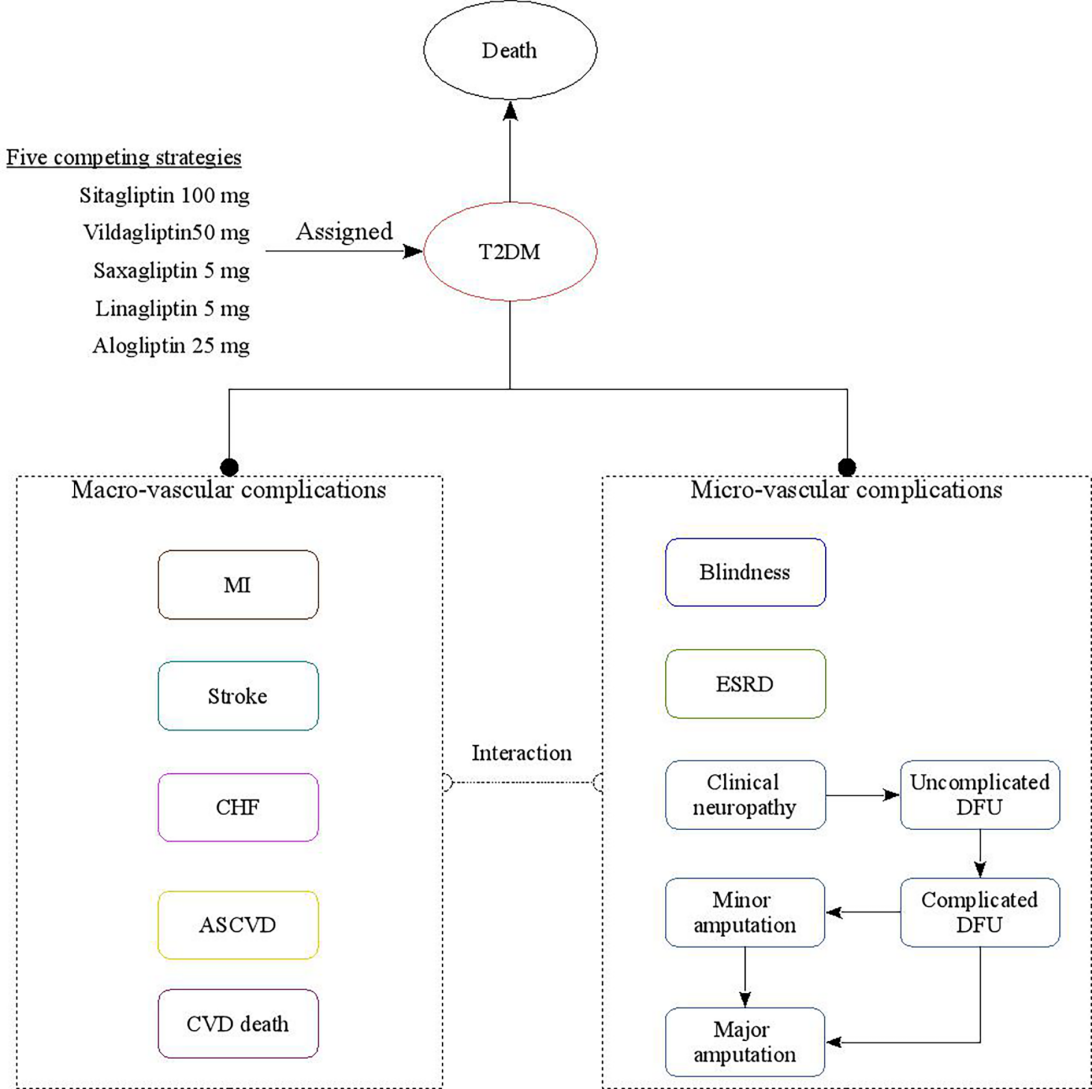
The structure of the Chinese T2DM health policy model.

The subjects of this study are Chinese patients with T2DM aged between 50 and 60 who were additively treated with DPP-4 inhibitors in the face of poor metformin monotherapy response. This study evaluated the economics of patients receiving five different strategies for DPP-4 inhibitors (including linagliptin 5 mg, saxagliptin 5 mg, alogliptin 25 mg, sitagliptin 100 mg, and vildagliptin 50 mg strategies). The analysis was conducted using a validated China diabetes policy analysis model called COMT (17–21) (**Figure 1**); this model can simulate a series of complications during treatment of a T2DM patient, including myocardial infarction (MI), congestive heart failure (CHF), cardiovascular disease (CVD), stroke, blindness, end-stage renal disease (ESRD), clinical neuropathy, foot ulcer, and minor and major amputation. The time horizon of the model is lifetime. All-cause mortality will be adjusted based on the treatment effect and disease status; long-term mortality is adjusted for the risk of all-cause death from diabetes. After entering the COMT model, patients will be assigned to different states according to diabetes complications. Transition probabilities in the model are derived from published literature. During the model simulation, interconnectivity and interaction among submodels of individual complication were permitted to allow the complication risks to be updated by using tracker parameters. The annual disease progression of the hypothetical cohorts with T2DM is determined based on demographic characteristics, disease history, disease-related clinical indicators, drug use history, etc. During the simulation, risk parameters might be adjusted based on the treatment transition, thereby resulting in the likelihood of complication incidence.

To keep with other economic reports associated with T2DM therapies (22), the primary outputs of the model included costs, cumulative probabilities of diabetic complications, life year (LY), quality-adjusted life year (QALY), and incremental cost-effectiveness ratios (ICERs). All costs and health output are discounted at a discount rate of 5%. In accordance with the recommendations of the World Health Organization (23) China’s per capita GDP in 2019 is used as the threshold ($10,276) in this study.

### Patient Profile and Treatment Effects

The patient characteristic profiles of receiving DPP-4 inhibitors were assumed to be similar to the SMART trial, which was an open-label Phase IV study comparing saxagliptin with acarbose in 488 Chinese patients with T2DM inadequately controlled with metformin monotherapy (24). When data pertaining to a specific parameter that was used for estimating the complications (25), such as history of smoking and anticoagulation usage, were not available, information from Chinese national cross-sectional were used as a reference (26). In the literature review by searching PubMed and Web of Knowledge, we electronically searched randomized controlled trials (≥24 weeks) including sitagliptin, vildagliptin, saxagliptin, linagliptin, and alogliptin that were published up to March 1, 2020. However, no head-to-head comparisons of sitagliptin, vildagliptin, saxagliptin, linagliptin, and alogliptin were reached. Thus, the clinical data was derived from a Bayesian mixed treatment comparison meta-analyses, which synthesized the treatment efficacy data reported by clinical trials (13). In this mixed treatment comparisons, the weighted absolute HbA1c changes from baseline (95% confidence interval) were −0.99 (−1.17 to −0.82), −1.03 (−1.21 to −0.85), −1.10 (−1.38 to −0.82), −1.06 (−1.22 to −0.91), and −1.02 (−1.18 to −0.86) in linagliptin 5 mg, saxagliptin 5 mg, alogliptin 25 mg, sitagliptin 100 mg, and vildagliptin 50 mg strategies, respectively. The mean absolute changes from baseline in HbA1c levels were employed in the first year of therapy. In the subsequent year, HbA1c was mimicked to rise naturally (nonlinear fashion) due to progressive nature of the disease, according to the HbA1c trajectories analysis. Because the adverse events with gliptin treatment, such as hypoglycemia, were at placebo level (27, 28), the current analysis did not consider the cost and disutilities related to adverse events.

### Costs and Utilities

Cost estimation from the perspective of China Health System shows that the study mainly addresses direct medical costs related to T2DM and its complications (**Table 1**). All cost data are discounted to 2019, shown as 2019 US dollars (1 US $ = 7.0 Chinese Yuan). The prices of sitagliptin, vildagliptin, saxagliptin, linagliptin, and alogliptin were derived from local Chinese database. The treatment regimens were administered as alogliptin 25 mg QD, linagliptin 5 mg QD, saxagliptin 5 mg QD, sitagliptin 100 mg/day, and vildagliptin 50 mg BID, respectively. The cost of antidiabetic treatment and blood glucose test strips related to T2DM was collected from a large screening study based on the national population, which interviewed 1,482 adult patients with T2DM in China (29). The costs of adverse events, including hypoglycemic events were derived from Chinese cost studies (30, 38). Other potential health resource consumption, such as outpatient and hospital expenses related to diabetes complications, are directly extracted from published literature or other local sources (30–35).

**Table 1:** Key model inputs of costs and utilities.

Parameters	Expected value	Range	Source
Costs ($)			
Sitagliptin 100 mg	1.05	0.53–1.05	Local charge
Vildagliptin 50 mg	1.17	0.58–1.17	Local charge
Saxagliptin 5 mg	1.14	0.57–1.14	Local charge
Linagliptin 5 mg	1.17	0.59–1.17	Local charge
Alogliptin 25 mg	1.15	0.58–1.15	Local charge
Antidiabetic therapy per day (disease duration ≤3 years)	0.5	0.2–1.3	(29)
Antidiabetic therapy per day (3 < disease duration ≤5 years)	0.8	0.2–1.7	(29)
Antidiabetic therapy per day (5 < disease duration < 10 years)	1.2	0.3–2.5	(29)
Antidiabetic therapy per day (disease duration ≥10 years)	2.0	0.7–3.2	(29)
MI hospitalization per event	7,383.0	6505.2–8260.9	(30–33)
Care after MI per year	455.4	288.6–622.2	(30–33)
Stroke hospitalization per event	2,875.2	2184.6–4738.3	(30–33)
Care after stroke per year	506.9	445.9–828	(30–33)
CHF per year	1,507.7	1254.6–2632.3	(30–33)
ESRD per year	13,803.2	13153.8–14569.2	(34)
Blindness per year	1,642.0	1430.4–1853.5	(30–33)
Clinical neuropathy per month	60.9	26.2–101.4	(35)
Uncomplicated DFU per event	76.2	0–226.2	(35)
Complicated DFU per event	2,293.3	1228.5–2880.8	(35)
Minor amputation per event	3,316.9	2165.2–5038.9	(35)
Major amputation per event	5,019.2	2981.1–7738.2	(35)
Care after major amputation per month	338.1	0–600.7	(35)
Urinary tract infections per event	31.00	23.3–38.8	(30)
Genital infections per event	31.00	23.3–38.8	(30)
Health utility scores			
T2DM without complications	0.936	0.736–1	(36, 37)
Health disutility scores			
Stroke hospitalization for one month	1.000	0.236–1	(36)
Stroke after discharge	0.114	0.026–0.446	(36, 37)
MI hospitalization for one month	1.000	0.326–1	(36)
MI after discharge	0.170	0.036–0.616	(36, 37)
CHF	0.250	0.026–0.446	(36, 37)
ESRD	0.156	0.19–0.61	(30, 31, 35−34, 37)
Blindness	0.113	0.007–0.307	(30, 31, 35−34, 37)
Clinical neuropathy	0.090	0.985–0.645	(36, 37)
Uncomplicated DFU	0.250	0.213–0.287	(30–33, 35)
Complicated DFU	0.300	0.165–0.435	(30–33, 35)
Minor amputation	0.320	0.204–0.436	(30–33, 35)
Major amputation	0.380	0.264–0.496	(30–33, 35)
Discount rate	5%	3%–8%	

MI, myocardial infarction; CHF, congestive heart failure; CVD, cardiovascular disease; ESRD, end-stage renal disease; DFU, diabetic foot ulcer.

The Health Utility Score was collected from a report of 12,583 Chinese patients with T2DM, and a validated Chinese EQ-5D-5L instrument was used to investigate the health status utility score for diabetes mellitus, neuropathy, and cardio-cerebovascular disease without complications (36, 37). Other utility scores that were not reported in this study, such as ESRD and minor and major amputations, were retrieved from published studies (30–35, 37).

### Sensitivity Analyses

The impact of parameter uncertainty was explored by one-way sensitivity analysis on each model parameter. Results of the one-way sensitivity analysis were expressed as tornado charts. The ranges of the parameters used in the one-way sensitivity analyses were obtained from the published literature (**Table 1**). When reported data were not available, a range of ±25% of the base-case value was used. Random values were drawn from the chosen distributions as a second-order Monte-Carlo simulation of 1,000 patients to estimate the mean and 95% confidential intervals (CI) of costs and life-years gained. Beta distribution were attached to the probability, proportions, and utility and disutility scores; triangle distribution to cost estimates; and normal distribution to hazard ratio and patient characteristic profile. A cost-effectiveness acceptability curve (CEAC) is constructed to summarize the uncertainty of cost-effectiveness evaluation under different thresholds of willingness to pay.

## Results

### Base-Case Outcomes

**Figure 2:**
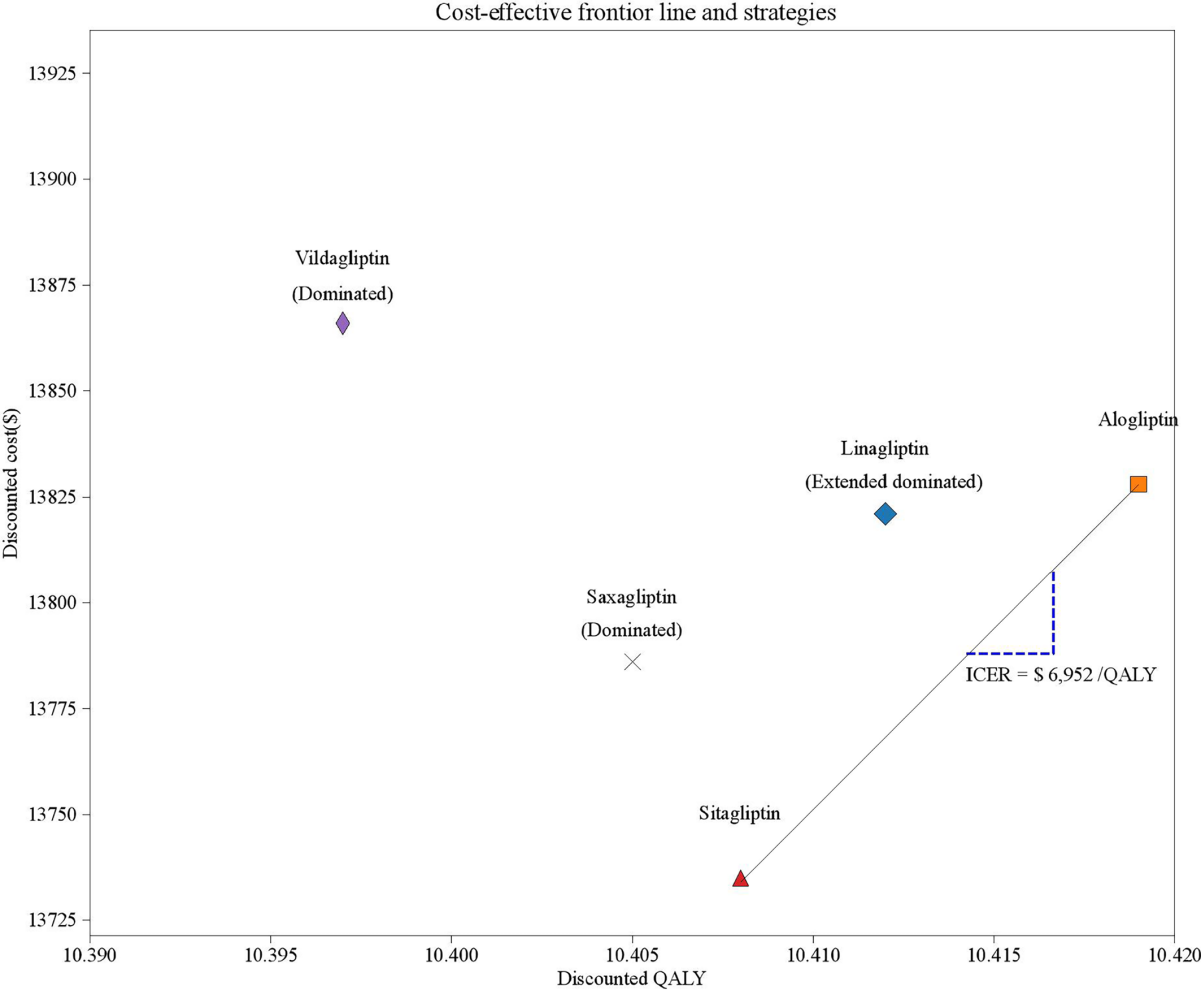
The cost-effectiveness efficiency frontier of five competing strategies.

Long-term projections of clinical outcomes indicated that alogliptin 25 mg was associated with improvements in LY of 0.014, 0.029, 0.022, and 0.044 years and improvements in discounted QALY of 0.007, 0.014, 0.011 and 0.022 QALYs *versus* linagliptin 5 mg, saxagliptin 5 mg, sitagliptin 100 mg, and vildagliptin50 mg, respectively (**Table 2**). The better health outcomes in the alogliptin 25 mg treatment arm arose from the reduced cumulative diabetes-related complications. In five competing strategies, sitagliptin 100 mg was associated with a cost of $13,735, which was lower than that of the other four strategies. Due to their relatively higher costs and lower health outcomes, both vildagliptin50 mg and saxagliptin 5 mg were dominated by the sitagliptin 100 mg strategies. The linagliptin 5 mg strategy was extended by the alogliptin 25 mg strategy. The cost-effectiveness efficiency frontier included the alogliptin 25 mg and sitagliptin 100 mg strategies (**Figure 2**).The ICER of alogliptin 25 mg against sitagliptin 100 mg strategy was $6,952 per additional QALY gained. The ICER is less than China’s per capita GDP in 2019, indicating that alogliptin 25 mg is cost-effective.

**Table 2:** Base-case results for five DDP-4 treatment strategies.

Outcomes	Linagliptin	Alogliptin	Saxagliptin	Sitagliptin	Vildagliptin
Events					
MI	9.63%	9.61%	9.65%	9.64%	9.67%
Stroke	22.06%	22.00%	22.13%	22.10%	22.19%
CHF	15.68%	15.65%	15.71%	15.69%	15.74%
ASCVD	15.44%	15.42%	15.46%	15.45%	15.49%
CVD death	22.41%	22.36%	22.46%	22.44%	22.51%
ESRD	4.14%	4.13%	4.15%	4.14%	4.15%
Blindness	4.13%	4.13%	4.13%	4.13%	4.13%
Clinical neuropathy	14.69%	14.69%	14.69%	14.69%	14.69%
Minor amputation	11.43%	11.43%	11.43%	11.43%	11.43%
Major amputation	8.43%	8.43%	8.43%	8.43%	8.43%
Total QALY	10.412	10.419	10.405	10.408	10.397
Total LY	20.852	20.866	20.837	20.844	20.822
Total Cost (US $)	13,821	13,828	13,786	13,735	13,866
ICER (US $/QALY)*	Extended dominated	6,952	Dominated	NA	Dominated

*Compared with the sitagliptin strategy because it is the cheapest strategy. NA, not applicable.

### Sensitivity Outcomes

**Figure 3:**
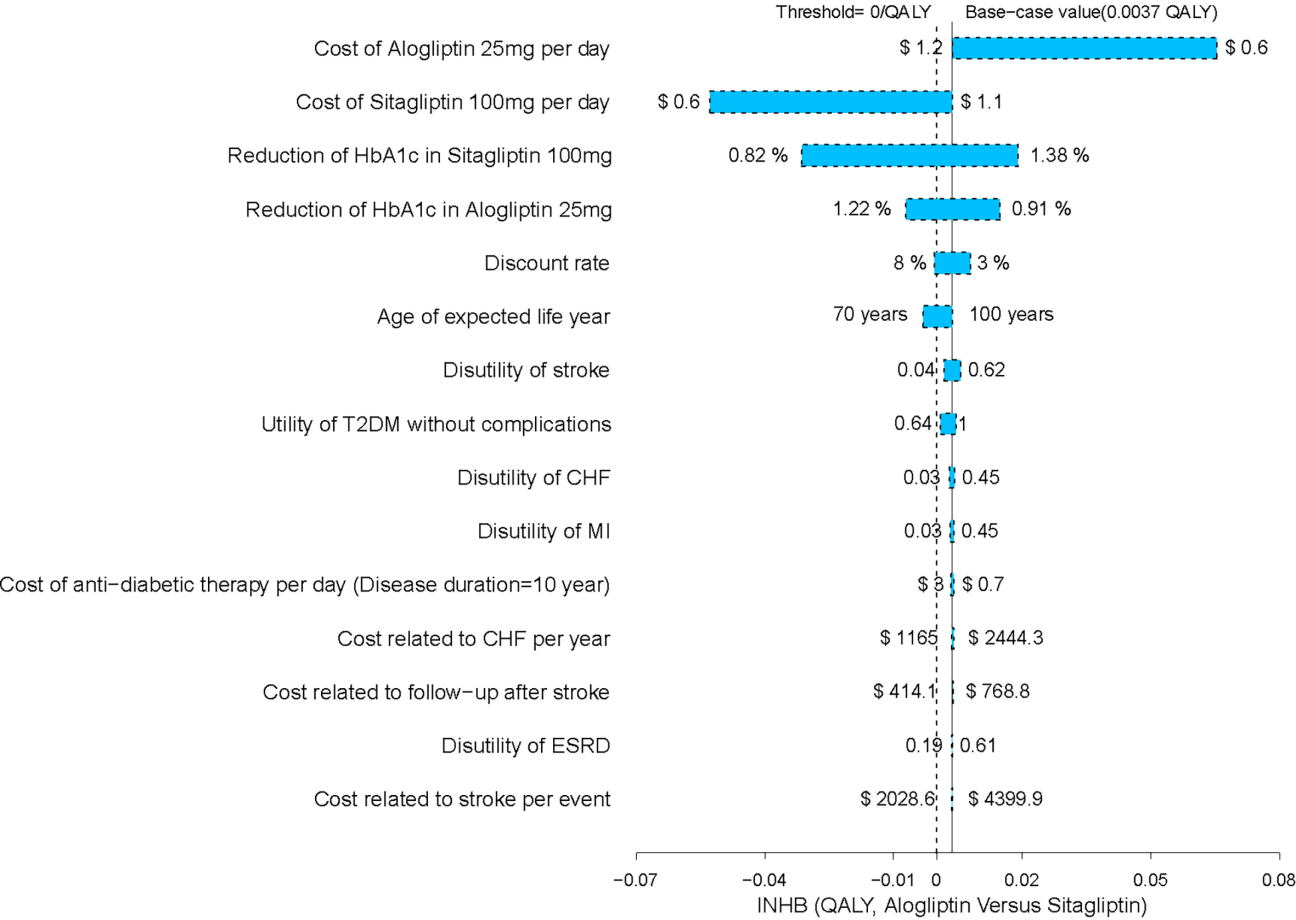
Tornado diagram for alogliptin *versus* sitagliptin strategy.

The one-way sensitivity analysis of alogliptin 25 mg against sitagliptin 100 mg strategy revealed that the results of the model were more sensitive to the cost of alogliptin and sitagliptin and the reductions in HbA1c in sitagliptin and alogliptin treatments. However, the variation does not exceed the threshold, that is, the result will not be reversed (**Figure 3**). We have also performed a one-way sensitivity analysis for the comparison of other drugs, and the results are robust.

**Figure 4:**
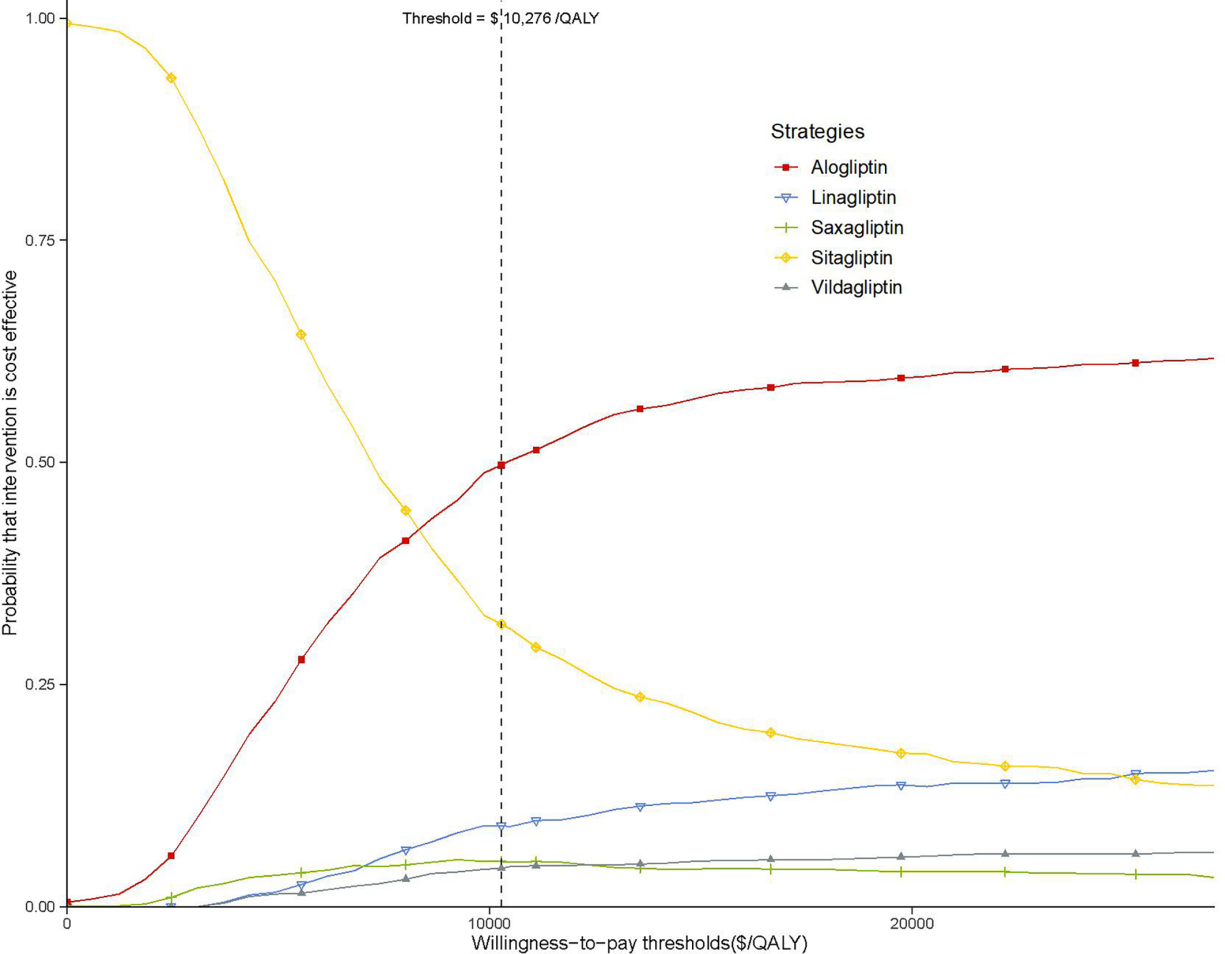
Acceptability curves comparing the cost-effectiveness of five competing strategies. The threshold is US$10,276 (the gross domestic product per capita of China in 2019).

As shown in the CEAC (**Figure 4**), the alogliptin 25 mg strategy produced a nearly half of the probability of cost-effectiveness compared with the other four competing strategies at an acceptable willingness-to-pay threshold of $10,276 (the GDP per capita of China in 2019).

## Discussion

Relevant clinical trials have shown that for those patients who have not reached the A1C target of metformin, in the context of metformin, the use of DPP-4 inhibitors as a second-line treatment is clinically effective. Due to the large number of T2DM patients in China and the limited health resources, the need to find the most cost-effective DPP-4 inhibitors has become urgent. The current economic analysis indicates that alogliptin 25 mg was a preferred treatment option as add-on to metformin in comparison with linagliptin 5 mg, saxagliptin 5 mg, sitagliptin 100 mg, and vildagliptin50 mg from the perspective of Chinese healthcare service providers. Compared with the other four DPP-4 inhibitors, alogliptin 25 mg has more health benefits and lower cost. As far as we know, this is the first cost-effectiveness analysis of the results of adding five DPP-4 inhibitors to adult T2DM patients with poor efficacy of metformin. One of the strengths of this study is the application of the COMT model, which shows good model validity for the established effects of interventions such as glucose and other intermediate endpoints in Chinese patients (17–21).

Several economic studies have reported the cost-effectiveness of DPP-4 inhibitors among adults with T2DM (15, 39–41). By using a third-party payer perspective of high- and middle-income countries and randomized clinical trials to measure effectiveness, sitagliptin, saxagliptin, and vildagliptin had an ICER below 25,000 €/QALY, as second-line and as add-ons to metformin, in comparison to sulfonylureas. An improvement in HbA1c, an intermediate biomarker, was indicated as a key driver of these cost-effective outcomes. Other economic evaluations also provided data to compare DPP-4 inhibitors *versus* insulin, and the results favored the use of DPP-4 inhibitors as second-line therapy (39). These findings generally show to be coherent across analytical models, payer perspective, and nations of analysis. However, there are few economic analyses that assessed the economic outcomes among DPP-4 inhibitors. In the Japanese context, a pharmacoeconomic analysis suggested that vildagliptin provides a superior cost–benefit compared with sitagliptin and alogliptin (42), which was distinguished with ours. The economic endpoint used in Teramachi and colleague’s study was the cost required for a 0.1% decrease in HbA1c for 12 weeks. This approach might not project the long-term outcomes. This might have contributed to the different findings of the present study, which used the lifetime COMT model and adopted the incremental cost per additional QALY gained as the endpoint.

A sensitivity analysis was conducted to identify the major influencing factors, verifying the stability of the model and the reliability of the advantage strategy. The results show that in the comparison between alogliptin and sitagliptin strategy that made up of the cost-effectiveness efficiency frontier, the most sensitive variables were the cost of alogliptin and sitagliptin. When the cost of alogliptin is reduced, the economic outcomes of alogliptin become more favorable. The reduction in the cost of alogliptin enlarges the gaps of incremental net health benefit (INHB) between sitagliptin and alogliptin strategy. However, when the cost of alogliptin is reduced, the INHB of sitagliptin might become lower than zero. The results from the probabilistic sensitivity analyses were similar to those of the base-case results, which produced relatively high probabilities of cost-effectiveness at the predefined Chinese threshold.

The current analysis has several limitations. First, given that there are no clinical trials that directly compare all DPP-4 inhibitors, the treatment effect data used in this study is the result of a Bayesian network meta-analysis (13). Fortunately, probability sensitivity analysis shows that the results of cost-effectiveness analysis are robust, and the uncertainty of each parameter will not have a significant impact on the results. Second, our results may only be applicable to T2DM patients in China but not to T2DM patients in other countries. All cost parameters are set for China and may be different from other countries. Third, due to the absence of available long-term health outcomes data of DPP-4 inhibitors as an add-on to metformin on the progression of diabetes-related complications, long-term comparisons may be uncertain. Finally, our decision analysis model is a simplification of actual disease results. The treatment strategy and clinical practice are based on Chinese guidelines and the recommendations of clinical metabolism experts; individual treatment decisions are not reflected in the model.

## Conclusion

This economic analysis found that alogliptin 25 mg strategy is likely to be more cost-effective in the Chinese healthcare system, as an alternative compared with linagliptin 5 mg, saxagliptin 5 mg, sitagliptin 100 mg, and vildagliptin 50 mg strategies for T2DM patients treated with DPP-4 inhibitors after failure of metformin treatment.

## Data Availability Statement

The original contributions presented in the study are included in the article/supplementary material.. Further inquiries can be directed to the corresponding authors.

## Ethics Statement

This study was based on literature review and modeling techniques; this study did not require approval by an institutional research ethics board.

## Author Contributions

WL and ZC contributed to the conception and design of the study; collecting data, analyzing and interpreting data, and drafting manuscripts. TC contributed to collecting data, analyzing and interpreting data. ML contributed to analyzing data and providing recommendations. BZ and NL contributed to the conception and design of the study. All authors contributed to the article and approved the submitted version.

## Funding

This study was supported by grants from Joint Funds for the innovation of science and Technology, Fujian province (grant number 2017Y9098), Science and Technology Department of Fujian Province (grant number 2020R0052), the National Natural Science Foundation of China (number 71373160), and Shanghai Municipal Health Commission (Evidence-based Public Health and Health Economics, number 15GWZK0901). The funding agency had no role in the study design, data collection and analysis, decision to publish, or preparation of the manuscript.

## Conflict of Interest

The authors declare that the research was conducted in the absence of any commercial or financial relationships that could be construed as a potential conflict of interest.

## Publisher’s Note

All claims expressed in this article are solely those of the authors and do not necessarily represent those of their affiliated organizations, or those of the publisher, the editors and the reviewers. Any product that may be evaluated in this article, or claim that may be made by its manufacturer, is not guaranteed or endorsed by the publisher.
